# Effects of perceptions of care, medical advice, and hospital quality on patient satisfaction after primary total knee replacement: A cross-sectional study

**DOI:** 10.1371/journal.pone.0178591

**Published:** 2017-06-13

**Authors:** Tom Schaal, Tonio Schoenfelder, Joerg Klewer, Joachim Kugler

**Affiliations:** 1 Department of Public Health, Dresden Medical School, University of Dresden, Loescherstrasse 18, Dresden, Saxony, Germany; 2 Department of Public Health and Health Care Management, University of Applied Sciences Zwickau, Dr.-Friedrichs-Ring 2A, Zwickau, Saxony, Germany; Public Library of Science, FRANCE

## Abstract

**Introduction:**

The increase in the number of patients presenting with osteoarthritis in the past decade has led to a 32% increase in knee replacement surgeries designed to reduce restrictions on patient movement and improve their quality of life. Patient satisfaction is becoming an increasingly important indicator of quality of care. This study was designed to identify predictors of various service components in the treatment process and hospital key performance indicators significantly associated with patient satisfaction.

**Materials and methods:**

A multicenter cross-sectional study was conducted with 856 patients having their primary total knee replacements at 41 hospitals. Patient satisfaction was queried via a validated, multidimensional questionnaire mainly using a six-point scale. In addition to bivariate calculations, patient satisfaction was the dependent variable in a binary logistic regression model.

**Results:**

The bivariate analysis showed a strong association between satisfaction and sex (male or female), the patients’ health before admission, and the length of stay. The number of cases treated at each hospital did not reveal any impact on satisfaction. The multivariate analysis identified three predictors associated with overall satisfaction. The strongest factor was the treatment outcome and the weakest was the quality of food. It became apparent that the statutory procedure minimums were not being met.

**Conclusions:**

The relevant factors influencing patient satisfaction were partially the same as previous study results and allowed more detailed conclusions. The results provide suggestions across hospitals that could help health care providers better meet needs of patients after knee arthroplasties.

## Introduction

Current concepts for the quality of care are no longer based solely on meeting evidence-based guidelines, but take into account information concerning patient satisfaction. These provide hospitals with indispensable information about their patients’ expectations and preferences which tend to focus on the interpersonal relationships in health care and the quality of service received and can serve as a basis for improving quality and competitiveness [1–2]. This is driven by health care reforms promoting competition in the marketplace and patients sensitive to differences in quality among hospitals who will change providers with better (perceived) quality [3]. Patient satisfaction is multidimensional and provides information about how services are perceived by patients and helps to isolate problem areas in hospitals and create appropriate solutions [4]. Satisfaction can be defined as the sufficient satisfaction of a patient’s needs and wishes with patients’ attributing varying levels of importance to their satisfaction [5].

Given the increased number of osteoarthritis cases, the demand for knee arthroplasties among elderly patients is climbing. These surgeries are designed to allow greater physical activity among patients who before surgery experience functional limitations and pain that conservative treatments have not been able to ameliorate [6–7]. In the industrialized countries alone, 27% of over 45-year-olds suffer from osteoarthritis. Total knee replacement (TKR) has the highest incidence. The OECD (Organisation for Economic Cooperation and Development) countries saw an increase from 114 to 150 knee replacement patients per 100,000 population between 2005 and 2011 [8–9].

As regulated in § 137 of the Code of Social Law, Book V, a minimum quantity of 50 total knee replacements per year and hospital has been required in Germany since 2006. These are specified in the minimum quantity regulation. The regulation identifies operative interventions in which the quality of the treatments outcome depends on the amount of operations performed [10–12]. Internationally, there is no consensus whether minimum quantities serve as a quality indicator or encourage negative after-effects [13–14]. Shan et al. [15] investigated 19 studies, in which over three-quarters of patients were overall satisfied with their knee replacement. Schulz & Scharf [16] examined 25 international studies and found that 84.3% (SD± 8.2) were overall satisfied with their knee replacement. Although patient satisfaction is difficult to measure and different instruments and scales are available, the factors most often studied to determine satisfaction with knee replacements include joint function, pain, mental wellbeing, and vitality [15–17]. Chang et al. [2] examined aspects of the service and medical received during inpatient joint replacements and came to the conclusion that, given the heterogeneous makeup of their patient populations, hospitals should identify those factors result in increased satisfaction among a majority of patients. The limited amount of published research in this fields has to date not allowed any generalizable statements to be made about the causal relationships behind satisfaction with arthroplasty, with multicenter studies recommended to better identify the predictors of satisfaction [15–16, 18].

Therefore, the aim of this study was to identify the medical and service-related parameters and hospital characteristics significantly related to patient satisfaction after TKR.

## Patients and methods

### Patient data and patient recruitment

The study data was collected via a self-administered questionnaire after the hospital stay. The questionnaire was based on a survey instrument developed on the basis of a review of patient satisfaction literature. Furthermore, published instruments and verbatim patient responses to questions about hospital quality provided by a health insurance provider. The questionnaire was drafted in German, construct validity has been tested by factor analysis [19]. This questionnaire was sent to patients’ home. The survey was conducted between February and June 2012. A cover letter informed the patients about the purpose of the survey; thus, participation in the study was voluntary and anonymous. By returning the questionnaire, patients gave their consent to participate in the study. Of 7,108 return postage-paid questionnaires mailed, 887 have been answered to and returned. Patients were excluded if the question of overall satisfaction remained unanswered or if no routine data were available for the respective hospital [20]. At the time of the survey, the treatment of patients had been completed for 1–25 months.

Selected randomly from orthopedic patients at 46 hospitals in a single German state, the study population consisted all received their primary total knee replacement in 2010–2011. The total population of this state was 3.06 million in 2011.

Based on the performance data of the health insurance providers, patients were contacted that had been billed with a DRG (Diagnosis Related Group) for primary total knee replacement. DRG is a classification system for a flat-rate billing procedure, which assigns hospital cases to medical case groups due to their methodological similarity [10]. The questionnaire was addressed to patients covered by statutory health insurance provided by five companies who together cover 78% of the total population. The insurance companies made the initial contact with the patients; it was not done directly by the hospitals to avoid any chance for biased selection of participants. In hospitals with more than 300 TKR cases a year, 300 patients were chosen at random, whereas in hospitals with less then 300 cases, all patients were involved. Study participants were randomly selected on basis of age, sex, and the market share of their health insurance provider of the federal state where the study was conducted. In total, a maximum of 600 patients were contacted per hospital for the years 2010 and 2011.

### Data collection

**Fig 1:**
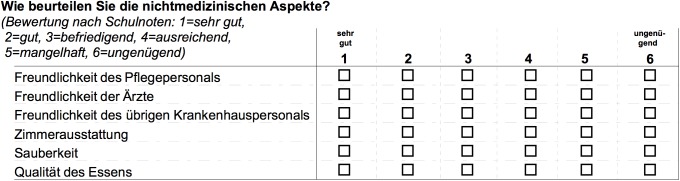
Excerpt of service-related questions of the questionnaire in German.

Patient satisfaction, socio-demographic information and information about their hospital stays were collected using the questionnaire [21–22]. Patient satisfaction was assessed using a set of questions related to medical care (10 questions) and service (6 questions, Fig 1) received, with each to be rated on a six-point scale (very good, good, satisfactory, adequate, inadequate, and unsatisfactory).

In addition, overall satisfaction with the hospital and the assessment of their treatment outcomes were also reported using the same scale. For further statistical analysis, the ratings for overall satisfaction and treatment outcomes were transferred into “satisfied” (very good, good) and “dissatisfied” (satisfactory to unsatisfactory).

For this purpose, patients were asked: If you were to evaluate the hospitalization received, how would you rate it? Other questions asked for information on age (10-year intervals from 21 to >80), sex (male or female), and state of health before admission (excellent, good, fair, or poor). The questionnaire also ascertained the qualifications of the admitting physician. The actual duration of the hospitalization versus the perceived duration of this time were assessed. Additionally, the occurrence of complications after discharge was assessed (Table 1).

**Table 1:** Relationships between patient and hospital variables in the study population and overall satisfaction (n = 856).

	Variable	Value	Respondents (%)	Satisfaction		p-Value
				rating*	range**	
*Questionnaire data*	Gender	Male	289 (33.8)	5.28	2–6	0.036^a^
		Female	554 (64.7)	5.18	1–6	
		No response	13 (1.5)			
	Age (years)	21–30	1 (0.1)	6.0	6–6	0.22^b^
		41–50	7 (0.8)	5.14	5–6	
		51–60	87 (10.2)	5.18	4–6	
		61–70	249 (29.1)	5.19	1–6	
		71–80	412 (48.1)	5.21	1–6	
		> 80	97 (11.3)	5.34	4–6	
		No response	3 (0.4)			
	Number of prior hospital stays***	1–2	557 (65.1)	5.24	1–6	0.158^b^
		3–5	245 (28.6)	5.2	2–6	
		> 5	35 (4.1)	5.0	3–6	
		No response	19 (2.2)			
	State of health prior to hospitalization	Excellent	7 (0.8)	6.0	6–6	< 0.001^b^
		Good	153 (17.9)	5.23	1–6	
		Fair	300 (35)	5.12	2–6	
		Poor	376 (43.9)	5.26	1–6	
		No response	20 (2.3)			
	Source of referral	General practitioner	83 (9.7)	5.19	1–6	0.817^b^
		Specialist	723 (84.5)	5.22	1–6	
		Self-referral	9 (1.1)	5.38	4–6	
		Emergency	8 (0.9)	5.14	3–6	
		No response	33 (3.9)			
	Length of stay	1–2 days	2 (0.2)	6.0	6–6	0.063^b^
		3–7 days	103 (12)	5.21	1–6	
		1–2 weeks	632 (73.8)	5.23	1–6	
		> 2 weeks	111 (13)	5.1	2–6	
		No response	8 (0.9)			
	Perceived length of stay	Absolutely appropriate	626 (73.1)	5.31	2–6	< 0.001^b^
		Could have been longer	135 (15.8)	4.97	1–6	
		Could have been shorter	11 (1.3)	4.67	3–6	
		I cannot judge	73 (8.5)	4.85	1–6	
		No response	11 (1.3)			
	Complications after discharge	Yes	167 (19.5)	4.73	1–6	< 0.001^c^
		No	664 (77.6)	5.32	1–6	
		No response	25 (2.9)			
*Hospital data*	Number of cases treated by hospital	High	435 (50.8)	5.21	1–6	0.739^c^
		Low	421 (49.2)	5.21	1–6	
		Median (range)	257 (7–696)			
	Postoperative mobility (percentage of cases treated)	High	361 (42.2)	5.24	2–6	0.424^c^
		Low	495 (57.8)	5.2	1–6	
		Median (range)	99.7 (53–100%)			
	Reoperation during study period	Yes	712 (83.2)	5.19	1–6	0.397^c^
		No	144 (16.8)	5.32	2–6	

*Grouped median

**The best ratings were given a score of 6 and the worst a score of 1

***Within the prior five years.

a. Mann-Whitney U test

b. Kruskal-Wallis test

c. Chi-squared test.

Hospital characteristics regarding TKR were obtained from the systematic hospital quality reports that each hospital is required to publish biannually. Of particular interest in these reports were the number of TKR inpatient cases, postoperative mobility (neutral-zero method), and whether there was any reoperative surgery during the period reported (yes/no). The neutral-zero method describes the range of motion of a joint in degrees of an angle around a certain axis. The overall rate of patients who achieved the required postoperative mobility until discharge from inpatient treatment was included as a feature of interest.

### Statistical analysis

Descriptive statistics and frequencies were calculated. To analyze the data delivered with the six-point scales, the best (most positive) reviews were given a score of 6 and the worst (least positive) a score of 1. The significance level for the entire study was set to p< .05. The data analysis was done using SPSS, version 20.0 (SPSS Inc., Chicago, IL, USA; S1 File).

The satisfaction scores were distributed left-skewed for better analysis, which is why non-parametric tests were calculated. Proceeding from the overall satisfaction level, potential differences in patient- and hospital-related variables were calculated using the Chi-squared test or Fisher’s exact test for small cell values and multiple group comparisons were calculated using the Kruskal-Wallis test. Hospital-related values were dichotomized (median split) based on the number of inpatient cases with TKR and postoperative mobility to allow for further analysis. The Mann-Whitney U test was used on the questions about the medical care and service received and to assess the success of treatment.

The binary logistic regression with inclusion process was chosen as a multivariate analysis method where all variables of the bivariate analysis were added with a significance level of p < .05 were included as predictors of overall satisfaction [18]. Given the sample size, this procedure was used to obtain a simple model with few degrees of freedom. The dependent variable was ‘overall satisfaction’, which was graphed dichotomously as “satisfied” and “dissatisfied”.

Assuming that missing values were missing at random, they were calculated using multiple imputation (iterative Markov Chain Monte Carlo method, 10 iterations) to calculate the logistic regression. All items collected were included [20, 23–24]. The question about the referring physician had the highest rate of missing responses (3.86%).

## Results

In all, 856 questionnaires from 41 hospitals were evaluated. 64.7% of the sample were female, 71–80 years of age, and had been admitted to hospital 1 to 2 times in the five years before the primary total knee replacement. 84.5% of patients were referred by a specialist and 9.7% by a general practitioner; 1.1% were self-referrals and 0.9% were admitted as the result of an emergency. 43.9% of the respondents indicated poor health before being admitted for surgery; another 35% said their health fair, while 17.9% reported good health and only 0.8% said their health had been excellent. Just under three-quarters of the patients reported hospital stays of between 1 and 2 weeks. 73.1% of participants considered the length of stay appropriate, while 15.8% felt it should have been longer and only 1.3% thought it should have been shorter. Patients were questioned about the actual length of stay as well as the perceived length of stay, because medical records from the respective hospitals were not available. 79% of the patients assessed their treatment outcome as very good or good, while 21% gave reported it as only satisfactory to unsatisfactory. Postoperative complications reported on the patients’ own accounts (self-reported) in the questionnaire were found in 19.5% of the study participants. Pain (n = 90), infection (n = 22) and wound healing disturbance (n = 10) were most frequently reported. In addition, swelling (n = 6), movement restrictions (n = 6), bleeding (n = 5), reimplantation (n = 4) and incisional hernias (n = 1) were reported.

The number of primary total knee replacements performed at each hospital ranged from 7 to 696 in 2010; 33 of the 41 hospitals reported reimplantations and 5 reported deaths associated with the procedure. In terms of post-operative mobility measured using the neutral-zero method, between 53 and 100% reported success (Table 1).

A total of 758 (88.6%) study participants rated the overall experience during the hospital stay as very good or good (grouped median score: 5.34). Patients were most satisfied with understandable explanations of anesthesia (5.54) and the friendliness of the nursing staff (5.49), followed by the friendliness of physicians (5.48). The worst ratings were given to the period after discharge (5.07) and understandable explanation of the newly prescribed medications (5.03).

Bivariate analysis of all 16 criteria related to the medical care and service received showed a statistically significant (p < .001) influence on patients’ overall total satisfaction (Table 2). The length of stay and self-reported complications after discharge (p < .001) and sex (p = .036) and the state of health prior to hospitalization (p < .001) were also associated with the dependent variable (Table 1). Male patients were more satisfied (5.28) than female ones (5.18). Patients who saw their length of stay as appropriate were more satisfied (5.31), than patients who found it too short (4.97) or too long (4.67) or those who did not respond to this question (4.85). While respondents with excellent health prior to hospitalization were the most satisfied (6.0), patients who assessed their prior health as poor were more satisfied (5.26) than those who had assessed it as fair (5.12). The respondents rated their overall satisfaction lower when there were self-reported complications after discharge (4.73 vs. 5.32). There was no difference between the dependent variable and the number of inpatient cases with TKR, postoperative mobility, or repeat operations during the study period. The same applies to patients age, the number of prior hospitalizations, the referring doctor, or length of stay (Table 1).

**Table 2:** Individual assessment of satisfaction on items related to medical care and service received.

Satisfaction criterion	All patients			Satisfied patients^1^			Dissatisfied patients^2^			p-Value*
	*n*	rating**	*range*^*3*^	*n*	rating**	*range*^*3*^	*n*	rating**	*range*^*3*^	
Organization of hospital admission	854	5.44	1–6	757	5.5	3–6	97	4.91	1–6	<0.001
Doctor’s knowledge of medical history and course of the disease	796	5.33	1–6	703	5.39	1–6	93	4.63	1–6	<0.001
Clear physician answers to patient questions	845	5.34	1–6	750	5.41	1–6	95	4.55	1–6	<0.001
Assessment of medical care received	846	5.25	1–6	749	5.35	2–6	97	4.24	1–6	<0.001
Clear explanation of surgery	847	5.47	1–6	751	5.54	1–6	96	4.7	2–6	<0.001
Clear explanation of anesthesia	849	5.54	1–6	754	5.6	1–6	95	4.92	2–6	<0.001
Clear explanation of medications to be taken	781	5.03	1–6	689	5.13	1–6	92	4.05	1–6	<0.001
Organization and conduct of tests	847	5.34	1–6	750	5.41	1–6	97	4.7	1–6	<0.001
Privacy during testing	825	5.35	2–6	732	5.41	3–6	93	4.78	2–6	<0.001
After-discharge preparations	823	5.07	1–6	728	5.17	1–6	95	4.08	1–6	<0.001
Friendliness of the nursing staff	848	5.49	2–6	754	5.56	4–6	94	4.69	2–6	<0.001
Friendliness of the doctors	848	5.48	2–6	754	5.55	4–6	94	4.73	2–6	<0.001
Friendliness of other hospital staff	835	5.31	3–6	740	5.38	4–6	95	4.69	3–6	<0.001
Room amenities	849	5.12	1–6	751	5.19	2–6	98	4.41	1–6	<0.001
Cleanliness	853	5.32	1–6	755	5.39	3–6	98	4.67	1–6	<0.001
Quality of food	852	5.20	1–6	754	5.27	2–6	98	4.46	1–6	<0.001
Treatment outcome	840	5.23	1–6	743	5.34	1–6	97	3.56	1–6	<0.001

* Difference between satisfied and dissatisfied patients, Mann-Whitney U test

**Grouped median

1 Overall satisfaction ranked very good or good; grouped median (mean rank)

2 Overall satisfaction ranked satisfactory, adequate, inadequate, dissatisfactory; grouped median (mean rank)

3 The best ratings were given a score of 6 and the worst a score of 1

Multivariate analysis revealed three variables associated with overall satisfaction (Nagelkerke-R^2^ = .664; Chi-Square Hosmer-Lemeshow Godness-of-fit-statistic = 3,43, 8 df, P = .882; 93,9% of cases were correctly classified). The strongest factor was the treatments outcome (odds ratio [OR]: 2.309, 95% confidence interval [CI]: 1.58–3.38) followed by the room amenities (OR: 2.1, CI: 1.25–3.52) and the weakest factor was the quality of the food (OR: 1.655, CI: 1.07–2.57). No variables from the questions about medical care could be adequately protected against random responses and they had no significant impact on overall satisfaction.

## Discussion

The study identified three predictors of overall satisfaction in patients undergoing their primary total knee replacements in hospital settings: treatment outcome, room amenities and quality of food. It is evident that the kinds of service that can be controlled by hospital management could have a stronger impact than demographics and hospital performance figures.

### Key findings

The 88.6% of the patients in this study who gave their hospital stays a good to very good rating was somewhat higher than the results in previous studies [15–17]. Possible causes for the slightly increased overall satisfaction may be seen in the past few years with more soft-tissue surgical procedures and the improvement of prosthesis design and mechanics. In relation to other joint replacement operations such as hip replacements, the proportion of dissatisfied patients remains high and further research into potential causes is justified [25–26].

The differences in the bivariate analysis showed discrepancies with other studies. While the variables related to medical care were equally associated with overall satisfaction, the independence of sex as a variable could not be confirmed [18, 27]. As in other studies, there was no influence on Patient age and satisfaction found in this study [18, 25]. The number of inpatient cases of a hospital with TKR did not reveal any impact on satisfaction. Statutory minimums for the number of surgeries performed as indicators of quality were not relevant to patient satisfaction; the number of cases treated did not allow any predictions of satisfaction. At the same time, it was shown that there were hospitals that did not meet the minimum number of primary total knee replacements, a fact which could negatively impact clinical quality indicators such as postoperative wound infections [12–14, 28].

The results of the multivariate analysis confirm that preclinical patient characteristics do not affect overall satisfaction levels [18]. Concerning the questions about medical care received, the previous findings on the positive impact of interpersonal interaction could not be confirmed, however, the independent variables of “room fittings” and “food quality” could be made more precise. Nevertheless, the relationship with the medical staff was reported better in the satisfied group, even if it was not statistically significant. The present study did not replicate the results of Chang et al. showing the highest increase in satisfaction being achieved by medical professionals. The lack of influence from other hospital staff on the dependent variable was, however, confirmed [2]. No association between the dissatisfaction of patients due to insufficient medical information was found, unlike in previous studies, and questions about the quality of medical consulting/advice showed no influence on satisfaction [27].

The results of this study show that hospitals can influence patient satisfaction based on the predictors identified. From the patients’ perspective, these represent relevant steps throughout the care process and provide incentives for the implementation of effective measures for improvement [4]. As hotel-like components of the hospital stay, the quality of the food and the room had a significant impact on the satisfaction of patients undergoing their primary knee replacement surgery. For some patients, these serve as surrogate indicators for assessing whether the proper diagnosis and treatment options have been offered by the medical staff [29–30]. The room as an important factor for many patients includes such aspects as privacy, discretion, and flexibility for visitors, a fact that hospital administrators should consider as potential predictors and incentives to increase satisfaction [31]. The strongest association between was that between overall satisfaction and treatment outcome (OR: 2.309, 95% CI: 1.58 to 3.38). The reason may be patients’ high expectations of being able to regain a high degree of physical activity after surgery and reduce their prior functional limitations and pain, all of which depend largely on the outcome [6–7]. In addition, the patients’ lack of knowledge of individual factors, which may influence the outcome (e.g. diabetes, immunosuppression, arterial hypertension), leads to false expectations. The expected outcome can be derived by considering comorbidities, or a complicated diagnosis and can serve the patient as an orientation in the context of patient education. In this way more time is spent on information and counseling, whereby the doctor-patient relationship can be improved and a common view of the possible outcome created [32].

In spite of the age of the data of 5 years at the time of analysis, the results are considered relevant. It is possible that fracture around the implant, infections, dislocation, or difference in leg length may still lead to complications. These complications can also influence the overall satisfaction [33–34]. The age distribution of patients was stable in all age groups of 2010 to 2014, with minimal vacillations. An influence of the factor 'age' on the results is not expected [35–36].

In contrast to previous findings, the use of multicenter (41 hospitals) approach in this study allowed suggestions for improving patient satisfaction to be made on a wider, cross-institutional basis that could impact a large number of patients [2, 25, 37]. Since the quality of medical care and service received has intangible aspects, the use of a structured, multidimensional questionnaire was shown able to identify a relationship between patient expectations and quantitatively-based treatment options.

### Limitations

When interpreting the results of this cross-sectional study, several limitations should be considered. First, no information was available about those who chose not to participate. Emberton & Black [38] came to the conclusion that non-respondents to post-surgery satisfaction are frequently older and in a worse state of health than those who did respond. The error of overestimating positive results and underestimating adverse effects by these individuals was nonetheless estimated to be low. Despite the tendency for worse ratings of satisfaction among non-respondents, Polk et al. [39] found that this does not affect the overall result. Lasek et al. [40] suggest that the impact of nonresponse bias on satisfaction surveys of hospitalized patients may be relatively small and that the effects were not systematically greater in hospitals with lower response rates. In summary, findings from satisfaction surveys should not be excluded from scientific contributions due to low response rates. Potential non-response bias and their resulting limitations of the study results cannot be excluded, because the influence of non-participants could not be controlled and the net rate of return was very low with 12.04% [25]. Maybe patients were more willing to take part in the survey if it was close to the treatment. The majority of participants were older people, in which functional or cognitive limitations may exist and complicate filling or understand the questionnaire [41]. A follow-up action by reminders or telephone inquiries could lead to a higher response rate, though it may be supposed that this will not fulfill with the anonymity guaranteed. Second, the sample of patients from 41 hospitals approximates the network of hospitals in Germany, but whether the results can be generalized to other regions and countries remains to be clarified [42]. Third, recall bias because of the time interval between treatment in 2010 and the questionnaire survey in 2012 can not be excluded, e.g. with regard to self-reported complications. Fourth, it's possible that satisfaction may increase after the TKR with gradual pain relief and restoration of function. With very satisfied patients, the contrary may occur as their expectations are increasing as a result of their good outcome, they may find that there is a ceiling to what they can achieve with a TKR from a functional point of view and there increasingly higher expectations may not be met [43]. There is also the possibility of selection bias if the sample of a heterogeneous population is not randomized stratified [44]. In the present study, the random sample was stratified by age, sex and market share of the health insurance providers. Surveys with regard to health insurance data may also lead to selection bias if they exclude a high proportion of non-insured persons [45]. In Germany, health insurances are part of the social security system and insurance obligation exists for all persons [10]. Thus, differences between insured persons and non-insured persons in the use of health services could almost be excluded. Therefore, the possibility of selection bias is considered to be low.

Nevertheless, with a variance of 66.4% explained with the Nagelkerke R^2^, the total satisfaction was well predicted by the independent variables and the Hosmer-Lemeshow test also showed an adequate goodness of fit [46]. In contrast to conventional methods, the multiple imputation allows asymptotically unbiased estimates of the missing values [47].

## Conclusions

The findings indicate that variables which dependent on health professionals and service personnel have an influence on satisfaction compared to other, fixed parameters. Improving patient education by doctors on achievable outcomes may prevent false expectations of the treatment. Service personnel can identify patients' wishes and contribute to an improvement in the quality of the food. This study identified predictors of overall satisfaction, which can be used to optimize processes and increase the satisfaction of patients having their primary knee replacement surgery.

## Declarations

### Ethics statement

In Germany, health insurance providers may carry out patient surveys to determine the quality of care without involving an ethics committee. This follows from §92 and §137 of the Social Code Book V (Sozialgesetzbuch V, SGB V), in conjunction with §4 of the Quality management guideline (Qualitätsmanagement-Richtlinie vertragsärztliche Versorgung, ÄQM-RL). A cover letter informed the patients about the purpose of the survey; thus, participation in the study was voluntary and anonymous. By returning the questionnaire, patients gave their consent to participate in the study. The survey was conducted in accordance with the ethical standards of the Declaration of Helsinki.

## Supporting information

S1 File Minimal data set. The file contains the original record for SPSS.
(SAV)

## References

[1] Porter ME. What Is Value in Health Care?. N Engl J Med. 2010;363(26):2477–81. doi: 10.1056/NEJMp1011024 21142528 10.1056/NEJMp1011024

[2] Chang CS, Chen SY, Lan YT. Service quality, trust, and patient satisfaction in interpersonal-based medical service encounters. BMC Health Serv Res. 2013;13(22). 10.1186/1472-6963-13-22 PMC3570322 23320786

[3] Varkevisser M, van der Geest SA, Schut FT. Do patients choose hospitals with high quality ratings? Empirical evidence from the market for angioplasty in the Netherlands. J Health Econ. 2012;31(2):371–8. doi: 10.1016/j.jhealeco.2012.02.001 22425770 10.1016/j.jhealeco.2012.02.001

[4] Sitzia J, Wood N. Patient satisfaction: A review of issues and concepts. Soc Sci Med. 1997;45(12):1829–43. 9447632 10.1016/s0277-9536(97)00128-7

[5] Crow R, Gage H, Hampson S, Hart J, Kimber A, Storey L, et al The measurement of satisfaction with healthcare: implications for practice from a systematic review of the literature. Health Technol Assess. 2002;6(32):1–244. 12925269 10.3310/hta6320

[6] Jones CA, Beaupre LA, Johnston DW, Suarez-Almazor ME. Total joint arthroplasties: current concepts of patient outcomes after surgery. Clin Geriatr Med. 2005;21(3):527–41. doi: 10.1016/j.cger.2005.02.005 15911205 10.1016/j.cger.2005.02.005

[7] Frankel L, Sanmartin C, Conner-Spady B, Marshall DA, Freeman-Collins L, Wall A, et al Osteoarthritis patients' perceptions of “appropriateness” for total joint replacement surgery. Osteoarthritis Cartilage. 2012;20(9):967–73. doi: 10.1016/j.joca.2012.05.008 22659599 10.1016/j.joca.2012.05.008

[8] Turkiewicz A, Petersson IF, Björk J, Hawker G, Dahlberg LE, Lohmander LS, et al Current and future impact of osteoarthritis on health care: a population-based study with projections to year 2032. Osteoarthritis Cartilage. 2014;22(11):1826–32. doi: 10.1016/j.joca.2014.07.015 25084132 10.1016/j.joca.2014.07.015

[9] Pabinger C, Lothaller H, Geissler A. Utilization rates of knee-arthroplasty in OECD countries. Osteoarthritis Cartilage. 2015;23(19):1664–73. 26028142 10.1016/j.joca.2015.05.008

[10] Code of Social Law, Book V (Das Fünfte Buch Sozialgesetzbuch–Gesetzliche Krankenversicherung), last amended on 17 July 2016.

[11] Minimum quantity regulation (Mindestmengenregelungen, Mm-R), last amended on 17 March 2016.

[12] Geraedts M, de Cruppé W, Blum K, Ohmann C. Implementation and Effects of Germany's Minimum Volume Regulations—Results of the Accompanying Research. Dtsch Arztebl Int. 2008;105(51–52):890–6. doi: 10.3238/arztebl.2008.0890 19561803 10.3238/arztebl.2008.0890 PMC2689625

[13] Halm EA, Lee C, Chassin MR. Is Volume Related to Outcome in Health Care? A Systematic Review and Methodologic Critique of the Literature. Ann Intern Med. 2002;137(6):511–20. 12230353 10.7326/0003-4819-137-6-200209170-00012

[14] Dudley RA, Johansen KL, Brand R, Rennie DJ, Milstein A. Selective referral to high-volume hospitals: estimating potentially avoidable deaths. JAMA. 2000;283(9):1159–66. 10703778 10.1001/jama.283.9.1159

[15] Shan L, Shan B, Suzuki A, Nouh F, Saxena A. Intermediate and Long-Term Quality of Life After Total Knee Replacement. A Systematic Review and Meta-Analysis. J Bone Joint Surg Am. 2015;97(2):156–68. doi: 10.2106/JBJS.M.00372 25609443 10.2106/JBJS.M.00372

[16] Schulze A, Scharf HP. Satisfaction after total knee arthroplasty. Comparison of 1990–1999 with 2000–2012. Orthopade. 2013; 42(10): 858–65. doi: 10.1007/s00132-013-2117-x 23695195 10.1007/s00132-013-2117-x

[17] Boniforti F, Macaione A, Gagliardi S, Giangrasso F, Di Marzo D, Giacco F. Early assessment of patient perception of outcome in total knee replacement. Joints. 2014;2(2):71–5. 25606546 10.11138/jts/2014.2.2.071 PMC4295672

[18] Baumann C, Rat AC, Osnowycz G, Mainard D, Delagoutte JP, Cuny C, et al Do clinical presentation and pre-operative quality of life predict satisfaction with care after total hip or knee replacement?. J Bone Joint Surg Br. 2006;88(3):366–73. doi: 10.1302/0301-620X.88B3.17025 16498013 10.1302/0301-620X.88B3.17025

[19] Schoenfelder T, Klewer J, Kugler J. Factors associated with patient satisfaction in surgery: the role of patients’ perceptions of received care, visit characteristics, and demographic variables. J Surg Res. 2010;164(1):e53–9. doi: 10.1016/j.jss.2010.08.001 20863521 10.1016/j.jss.2010.08.001

[20] Sterne JAC, White IR, Carlin JB, Spratt M, Royston P, Kenward MG, et al Multiple imputation for missing data in epidemiological and clinical research: potential and pitfalls. BMJ. 2009;338: doi: 10.1136/bmj.b2393 19564179 10.1136/bmj.b2393 PMC2714692

[21] Schoenfelder T, Klewer J, Kugler J. Determinants of patient satisfaction: A study among 39 hospitals in an in-patient setting in Germany. Int J Qual Health Care. 2011;23(5): 503–9. doi: 10.1093/intqhc/mzr038 21715557 10.1093/intqhc/mzr038

[22] Schoenfelder T, Schaal T, Klewer J, Kugler J. Patient Satisfaction in Urology: Effects of Hospital Characteristics, Demographic Data and Patients’ Perceptions of received care. Urol J. 2014;11(4):1834–40. 25194086

[23] Gandhi R, Dhotar H, Tso P, Davey R, Mahomed NN. Predicting the longer term outcomes of total knee arthroplasty. Knee. 2010;17(1):15–8. doi: 10.1016/j.knee.2009.06.003 19589683 10.1016/j.knee.2009.06.003

[24] de Goeij MC, van Diepen M, Jager KJ, Tripepi G, Zoccali C, Dekker FW. Multiple imputation: dealing with missing data. Nephrol Dial Transplant. 2013;28(10):2415–20. doi: 10.1093/ndt/gft221 23729490 10.1093/ndt/gft221

[25] Bamm EL, Rosenbaum P, Stratford P. Validation of the measure of processes of care for adults: a measure of client-centred care. Int J Qual Health Care. 2010;22(4):302–9. doi: 10.1093/intqhc/mzq031 20543207 10.1093/intqhc/mzq031

[26] Anakwe RE, Jenkins PE, Moran M. Predicting Dissatisfaction After Total Hip Arthroplasty: A Study of 850 Patients. J Arthroplasty. 2011;26(2):209–13. doi: 10.1016/j.arth.2010.03.013 20462736 10.1016/j.arth.2010.03.013

[27] Mann C, Gooberman-Hill R. Health Care Provision for Osteoarthritis: Concordance Between What Patients Would Like and What Health Professionals Think They Should Have. Arthritis Care Res. 2011;63(7):963–72. 10.1002/acr.20459 21387574

[28] Meredith DS, Katz JN. Procedure volume as a quality measure for total joint replacement. Clin Exp Rheumatol. 2007;25(47):37–43. 18021505

[29] Otani K, Kurz RS, Harris LE. Managing primary care using patient satisfaction measures. J Healthc Manag. 2005;50(5):311–24. 16268410

[30] Asadi-Lari C, Packham C, Gray D. Is quality of life measurement likely to be a proxy for health needs assessment in patients with coronary artery disease?. Health Qual Life Outcomes. 2003;1(50). 10.1186/1477-7525-1-50 PMC240110 14596682

[31] Maben J, Griffiths P, Penfold C, Simon M, Anderson JE, Robert G, et al One size fits all? Mixed methods evaluation of the impact of 100% single-room accommodation on staff and patient experience, safety and costs. BMJ Qual Saf. 2015; doi: 10.1136/bmjqs-2015-004265 26408568 10.1136/bmjqs-2015-004265 PMC4819646

[32] Alami S, Boutron I, Desjeux D, Hirschhorn M, Meric G, et al Patients’ and Practitioners’ Views of Knee Osteoarthritis and Its Management: A Qualitative Interview Study. PLoS ONE. 2011;6(5): e19634 doi: 10.1371/journal.pone.0019634 21573185 10.1371/journal.pone.0019634 PMC3088707

[33] Kenney NA, Farmer KW. Minimally invasive versus conventional joint arthroplasty. PM R. 2012;4(5 Suppl):134–40. 10.1016/j.pmrj.2012.01.006 22632692

[34] Meier E, Gelse K, Trieb K, Pachowsky M, Hennig FF, Mauerer A. First clinical study of a novel complete metal-free ceramic total knee replacement system. J Orthop Surg Res. 2016;11(21): doi: 10.1186/s13018-016-0352-7 26857704 10.1186/s13018-016-0352-7 PMC4745159

[35] AQUA–Institut für angewandte Qualitätsförderung und Forschung im Gesundheitswesen. Bundesauswertung zum Erfassungsjahr 2011: 17/5 –Knie-Totalendoprothesen-Erstimplantation. Goettingen, 2012.

[36] AQUA–Institut für angewandte Qualitätsförderung und Forschung im Gesundheitswesen. Bundesauswertung zum Erfassungsjahr 2014: 17/5 –Knie-Totalendoprothesen-Erstimplantation. Goettingen, 2015.

[37] Barlow T, Dunbar M, Sprowson A, Parsons N, Griffin D. Development of an outcome prediction tool for patients considering a total knee replacement–the Knee Outcome Prediction Study (KOPS). BMC Musculoskelet Disord. 2014;15(451). 10.1186/1471-2474-15-451 PMC4364581 25539734

[38] Emberton M, Black N. Impact of non-response and of late-response by patients in a multi-centre surgical outcome audit. Int J Qual Health Care. 1995;7(1):47–55. 7640918 10.1093/intqhc/7.1.47

[39] Polk A, Rasmussen JV, Brorson S, Olsen BS. Reliability of patient-reported functional outcome in a joint replacement registry. Acta Orthop. 2013;84(1):12–7. doi: 10.3109/17453674.2013.765622 23343374 10.3109/17453674.2013.765622 PMC3584596

[40] Lasek RJ, Barkley W, Harper DL, Rosenthal GE. An evaluation of the impact of nonresponse bias on patient satisfaction surveys. Med Care. 1997;35(6):646–52. 9191708 10.1097/00005650-199706000-00009

[41] Gayet-Ageron A, Agoritsas T, Schiesari L, Kolly V, Perneger TV. Barriers to participation in a patient satisfaction survey: Who are we missing? PLoS ONE. 2011;6(10): e26852 doi: 10.1371/journal.pone.0026852 22046382 10.1371/journal.pone.0026852 PMC3202588

[42] Klit J, Jacobsen S, Rosenlund S, Sonne-Holm S, Troelsen A. Total Knee Arthroplasty in Younger Patients Evaluated by Alternative Outcome Measures. J Arthroplasty. 2013;29(5):912–7. doi: 10.1016/j.arth.2013.09.035 24269097 10.1016/j.arth.2013.09.035

[43] Lošťák J, Gallo J, Zapletalová J. Patient satisfaction after total knee arthroplasty. Analysis of pre-operative and peri-operative parameters influencing results in 826 patients. Acta Chir Orthop Traumatol Cech. 2016;83(2):94–101. 27167423

[44] Berk RA. An introduction to sample selection bias in sociological data. American Sociological Review. 1983;48(3):386–98.

[45] Heyden J, Charafeddine R, De Bacquer D, Tafforeau J, Herck K. Regional differences in the validity of self-reported use of health care in Belgium: selection versus reporting bias. BMC Med Res Methodol. 2016;16(98). 10.1186/s12874-016-0198-z PMC4986374 27528010

[46] Hosmer DW, Hosmer T, Cessie SL, Lemeshow S. A comparison of goodness-of-fit tests for the logistic regression model. Stat Med. 1997;15(9):965–80. 10.1002/(sici)1097-0258(19970515)16:9<965::aid-sim509>3.0.co;2-o 9160492

[47] Klebanoff MA, Cole SR. Use of Multiple Imputation in the Epidemiologic Literature. Am J Epidemiol. 2008;168(4):355–7. doi: 10.1093/aje/kwn071 18591202 10.1093/aje/kwn071 PMC2561989

